# Comparison of Different Neostigmine Doses for Reversal of Cisatracurium‐Induced Neuromuscular Block in Children Under Total Intravenous Anesthesia: A Randomized Controlled Trial

**DOI:** 10.1002/pan.70216

**Published:** 2026-05-08

**Authors:** Antonio J. M. M. Neto, Guilherme L. Benette, Letícia C. Siqueira, Marian M. Santos, Marco A. P. Costa, Rodrigo L. Alves, Eduardo T. Moro, Norma S. P. Módolo

**Affiliations:** ^1^ Department of Surgery Pontifical Catholic University of São Paulo (PUC‐SP), School of Medical and Health Sciences São Paulo Brazil; ^2^ Department of Surgical Specialties and Anesthesiology São Paulo State University (UNESP), Medical School São Paulo Brazil; ^3^ Department of Anesthesiology and Surgery Bahia School of Medicine, Federal University of Bahia Salvador Brazil

**Keywords:** anesthesia, monitoring, neostigmine, neuromuscular, neuromuscular blockade, pediatric

## Abstract

**Background:**

Neostigmine is widely used to reverse nondepolarizing neuromuscular blockade in children, but the optimal dose under total intravenous anesthesia is uncertain.

**Aims:**

The primary aim was to compare the time to full neuromuscular recovery (TOF ratio of 1.0) following administration of neostigmine at doses of 0, 10, 20, and 30 μg/kg in children at a TOF count of 3. Secondary objectives were full reversal within 10 min and adverse events.

**Methods:**

This prospective, randomized, double‐blind, parallel‐group, superiority trial enrolled 120 children (2–10 years; ASA I–II) undergoing tonsillectomy. Participants received 0, 10, 20, or 30 μg/kg neostigmine at a TOF count of 3 measured by quantitative acceleromyography. The primary outcome was the time from TOF count of 3 to full reversal (TOF ratio = 1.0). Secondary outcomes were the proportion of patients achieving full reversal within 10 min and adverse events. Comparisons among active groups used the Kruskal–Wallis test.

**Results:**

A total of 118 patients were analyzed. Median [IQR] time to full reversal was 20.2 [14.8–24.1], 14.0 [10.7–16.8], 11.0 [8.2–15.5], and 11.2 [7.9–14.6] min in the 0, 10, 20, and 30 μg/kg groups, respectively. Reversal was significantly slower in the control group compared with all neostigmine doses. However, there was no statistically significant difference among the active doses (Kruskal–Wallis, *p* = 0.33). At 10 min, full reversal had occurred in 10.7%, 23.3%, 43.3%, and 33.3% of patients in the respective groups. Adverse events were uncommon, occurring in 10 of 118 patients, and consisted exclusively of transient bradycardia and tachycardia, without differences among groups.

**Conclusions:**

At TOF count of 3, neostigmine 10–30 μg/kg shortened reversal compared with no reversal, but doses above 10 μg/kg conferred no additional benefit. Quantitative monitoring remains essential, as fewer than half of patients achieved a TOF ratio of 1.0 within 10 min.

**Trial Registry:**

https://ensaiosclinicos.gov.br/rg/RBR‐4xrx2g3

## 1 Introduction

General anesthesia is one of the most commonly used techniques worldwide, and the introduction of neuromuscular blockers represented a major advance in anesthetic practice, improving, among other aspects, airway management, surgical conditions, and respiratory outcomes [1, 2]. Although these benefits are well established, inadequate reversal remains clinically relevant, with residual neuromuscular block reported in up to 48.2% of pediatric patients at the time of extubation [3].

The efficacy of neostigmine depends on the degree of neuromuscular recovery at the time of administration [4]. Therefore, identifying its optimal dose and timing remains clinically important, particularly in children, whose recovery differs substantially from that of adults [5]. Neostigmine is widely available in clinical practice. It is safe, inexpensive, and, when properly administered, can provide outcomes comparable to those of newer reversal agents [6, 7].

Most studies to date have been conducted in adults, and to our knowledge, none have evaluated neostigmine under total intravenous anesthesia in children [8], a condition that does not potentiate neuromuscular block, as volatile anesthetics do. There remains a need to improve understanding and awareness regarding the safe, effective use and timely reversal of residual paralysis to enhance perioperative quality of care [9].

The primary aim of this randomized trial was to compare the effect of different neostigmine doses for recovery from moderate neuromuscular block in children under total intravenous anesthesia. Secondary aims included the proportion of patients achieving full reversal and the occurrence of adverse events.

## 2 Methods

This was a prospective, randomized, double‐blind, parallel‐group, superiority clinical trial. A total of 120 pediatric patients, aged 2 to 10 years and classified as American Society of Anesthesiologists (ASA) physical status I–II, undergoing elective tonsillectomy under general anesthesia, were recruited between April 2024 and September 2025. The study was conducted at Hospital Santa Lucinda in Sorocaba, Brazil, after approval by the Research Ethics Committee (CAAE 76362223.6.0000.5373, February, 2024) and registration in the Brazilian Clinical Trials Registry (RBR‐4xrx2g3, https://ensaiosclinicos.gov.br/rg/RBR‐4xrx2g3, April, 2024). Exclusion criteria included refusal to participate, renal, hepatic, or neuromuscular disease, contraindication to any of the study drugs, body mass index greater than 30 kg/m [2], or acute clinical illness. After randomization, patients were excluded in cases of surgical modification, excessive blood loss, or equipment malfunction.

The primary outcome was the time elapsed from a TOF count of 3 to a TOF ratio of 1.0. Secondary outcomes included: (1) the incidence of adverse events—patients were systematically assessed in the operating room and post‐anesthesia care unit for at least 60 min for nausea, vomiting, bradycardia (< 60 bpm), tachycardia (> 130 bpm), bronchospasm (wheezing with increased airway pressures), laryngospasm (stridor or complete absence of airflow), and oxygen desaturation (SpO_2_ < 93% despite supplemental oxygen); and (2) the proportion of patients achieving TOF ratio of 1.0 within 10 min after neostigmine administration. The TOF count represents the number of responses (0–4) to a train‐of‐four stimulation, whereas the TOF ratio is defined as the relation between the fourth and the first twitch. Bradycardia episodes, when deemed clinically relevant by the attending anesthesiologist, were treated with atropine 20 μg/kg.

Patients were assigned by computer‐generated simple randomization, evenly allocated (1:1:1:1) into four groups receiving 0, 10, 20, or 30 μg/kg of neostigmine with corresponding atropine doses of 0, 5, 10, and 15 μg/kg, respectively. Sealed opaque envelopes containing group allocations were sequentially numbered. An anesthesiologist not involved in the study prepared the solutions, diluted each to a total volume of 10 mL, and labeled them as “reversal.” The attending anesthesiologist responsible for intraoperative management, neuromuscular monitoring, and outcome assessment was unaware of group allocation and administered the study drug. Investigators and participants were blinded to group allocation. Written informed consent was obtained from parents or legal guardians; assent was also obtained from children aged over 6 years, in accordance with ethical recommendations.

De‐identified individual participant data and the statistical analysis plan will be made available upon reasonable request.

This manuscript adheres to the CONSORT guidelines.

### 2.1 Anesthesia

Monitoring included electrocardiography, noninvasive arterial pressure, pulse oximetry, and capnography. Intravenous access was obtained, but if not possible, inhaled anesthesia was induced prior to venipuncture using sevoflurane up to 6%. Anesthesia was induced and maintained continuously with propofol (150–200 μg/kg/min) and remifentanil (0.1–0.5 μg/kg/min), titrated according to clinical signs. Hemodynamic stability was maintained in all patients during the intraoperative period.

After a TOF count of zero was achieved with cisatracurium (0.1 mg/kg), as described below, orotracheal intubation was performed; mechanical ventilation was adjusted to normocapnia. According to the study protocol, additional doses of cisatracurium were administered if the TOF count reached three before completion of tonsillar excision. Axillary and thenar eminence temperatures were consistently maintained above 35°C and 32°C, respectively, using a warming blanket and cotton insulation. No adjuvant medications known to interfere with neuromuscular reversal were administered.

### 2.2 Neuromuscular Monitoring

Neuromuscular monitoring was initiated after loss of consciousness using an acceleromyograph (TOF‐Watch SX, Schering‐Plow, Swords, Dublin, Ireland), as recommended for clinical research [10]. The arm without a blood pressure cuff and intravenous access was chosen for monitoring. Fingers were fixed, and the sensor was attached to the volar side of the distal interphalangeal joint of the thumb, aligned with the axis of thumb movement. Pediatric electrodes were placed after skin preparation with alcohol swabs: the negative electrode was placed distally, and the positive electrode positioned proximally, 3–6 cm apart along the course of the ulnar nerve at the wrist, allowing monitoring of the adductor pollicis muscle. Calibration and supramaximal stimulation were obtained using the preprogrammed “CAL 2” mode on the device. At least 2 min of a stable signal (variations less than 5%) were required, after which the neuromuscular blocker was administered. Continuous TOF monitoring (four pulses of 0.2 milliseconds duration, frequency of 2 Hz, every 15 s) was recorded throughout surgery until tracheal extubation. Reversal was initiated only after a TOF count of 3 had remained stable for at least 2 min.

### 2.3 Statistical Analysis

Continuous variables were summarized as medians and interquartile ranges (IQRs) because of non‐normal distribution, and categorical variables as counts and percentages. Differences in baseline characteristics were assessed descriptively. The primary outcome, time to full reversal (TOF ratio = 1.0), was compared among the active neostigmine groups (10, 20, and 30 μg/kg) using the Kruskal–Wallis test. Pairwise comparisons were explored using the Hodges–Lehmann estimator of effect size with 95% bootstrap confidence intervals, chosen to provide robust estimates without assuming normality of the data. These comparisons were reported as effect sizes rather than hypothesis tests; therefore, multiplicity adjustment was not necessary. Cumulative probability of full reversal over time in minutes was illustrated with Kaplan–Meier curves. A two‐sided *p*‐value < 0.05 was considered statistically significant. Statistical analyses were performed using jamovi software (version 2.6.26; The jamovi Project, Sydney, Australia).

### 2.4 Sample Size Calculation

A pilot sample of 60 patients was used to estimate the variability of the primary outcome, conducted under the same protocol as the present study. The standard deviation of the time to full reversal was approximately 5 min. Assuming a clinically relevant difference of 5 min between groups, a two‐sided α of 0.05, and 80% power, the required sample size was 25 patients per group. To account for potential dropouts, we planned to include 30 patients in each group.

## 3 Results

**FIGURE 1:**
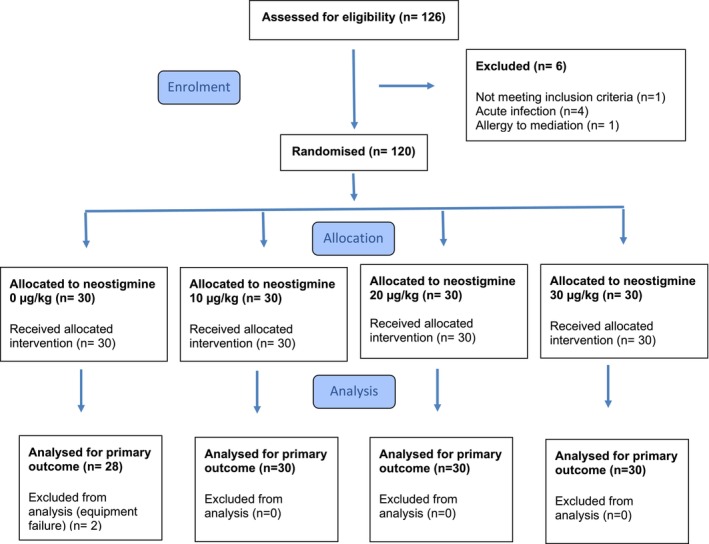
Flowchart of patient enrollment and analysis.

A total of 120 patients were randomized as shown in the flowchart (Figure 1), and 118 were included in the final analysis. Baseline characteristics were comparable across groups (Table 1). Propofol and remifentanil were administered as continuous infusions within the reported dose ranges, with no relevant differences in infusion ranges among the study groups.

**TABLE 1:** Baseline characteristics of participants according to neostigmine dose (data are presented as median [IQR], mean ± SD or n [%]).

Characteristic	0 μg/kg	10 μg/kg	20 μg/kg	30 μg/kg
Age (years)	5.0 [4.0–7.0]	6.0 [4.2–8.0]	6.0 [5.0–7.0]	6.0 [5.0–7.0]
Weight (kg)	22.0 [17.0–29.2]	24.5 [19.2–32.8]	23.0 [20.0–32.0]	23.0 [20.0–28.5]
Sex	Female: 14 (50.0%); Male: 14 (50.0%)	Male: 16 (53.3%); Female: 14 (46.7%)	Male: 17 (56.7%); Female: 13 (43.3%)	Male: 15 (50.0%); Female: 15 (50.0%)
ASA physical status	ASA I: 24 (85.7%); ASA II: 4 (14.3%)	ASA I: 22 (73.3%); ASA II: 8 (26.7%)	ASA I: 21 (70.0%); ASA II: 9 (30.0%)	ASA I: 27 (90.0%); ASA II: 3 (10.0%)

The median time to full neuromuscular recovery (TOF ratio = 1.0) was 20.2 min (IQR 14.8–24.1) in the control group (0 μg·kg^−1^), 14.0 min (IQR 10.7–16.8) in the 10 μg·kg^−1^ group, 11.0 min (IQR 8.2–15.5) in the 20 μg·kg^−1^ group, and 11.2 min (IQR 7.9–14.6) in the 30 μg·kg^−1^ group. The control group showed longer reversal times than all neostigmine groups. Among the active doses (10, 20, and 30 μg·kg^−1^), no clinically meaningful differences were detected (Kruskal–Wallis, *p* = 0.33). These results are summarized in Table 2. Pairwise comparisons, including contrasts between the control group and each neostigmine dose, were explored using the Hodges–Lehmann estimator with 95% bootstrap confidence intervals and are presented in Table 3.

**TABLE 2:** Time to full reversal (TOF ratio = 1.0).

Group (neostigmine dose)	*n*	Median [IQR] (min)
0 μg/kg	28	20.2 [14.8–24.1]
10 μg/kg	30	14.0 [10.7–16.8]
20 μg/kg	30	11.0 [8.2–15.5]
30 μg/kg	30	11.2 [7.9–14.6]

*Note:* No statistically significant differences were observed among the active neostigmine doses (10, 20, and 30 μg/kg; Kruskal–Wallis test, *p* = 0.33).

**TABLE 3:** Pairwise comparisons of time to full reversal.

Comparison (neostigmine dose)	Hodges–Lehmann difference (min)	95% confidence interval (min)
0 μg/kg vs. 10 μg/kg	6.11	2.85 to 9.58
0 μg/kg vs. 20 μg/kg	8.00	4.02 to 11.83
0 μg/kg vs. 30 μg/kg	8.05	4.17 to 11.17
10 μg/kg vs. 20 μg/kg	2.01	−0.63 to 4.73
10 μg/kg vs. 30 μg/kg	2.00	−0.42 to 4.12
20 μg/kg vs. 30 μg/kg	−0.08	−2.88 to 2.18

**FIGURE 2:**
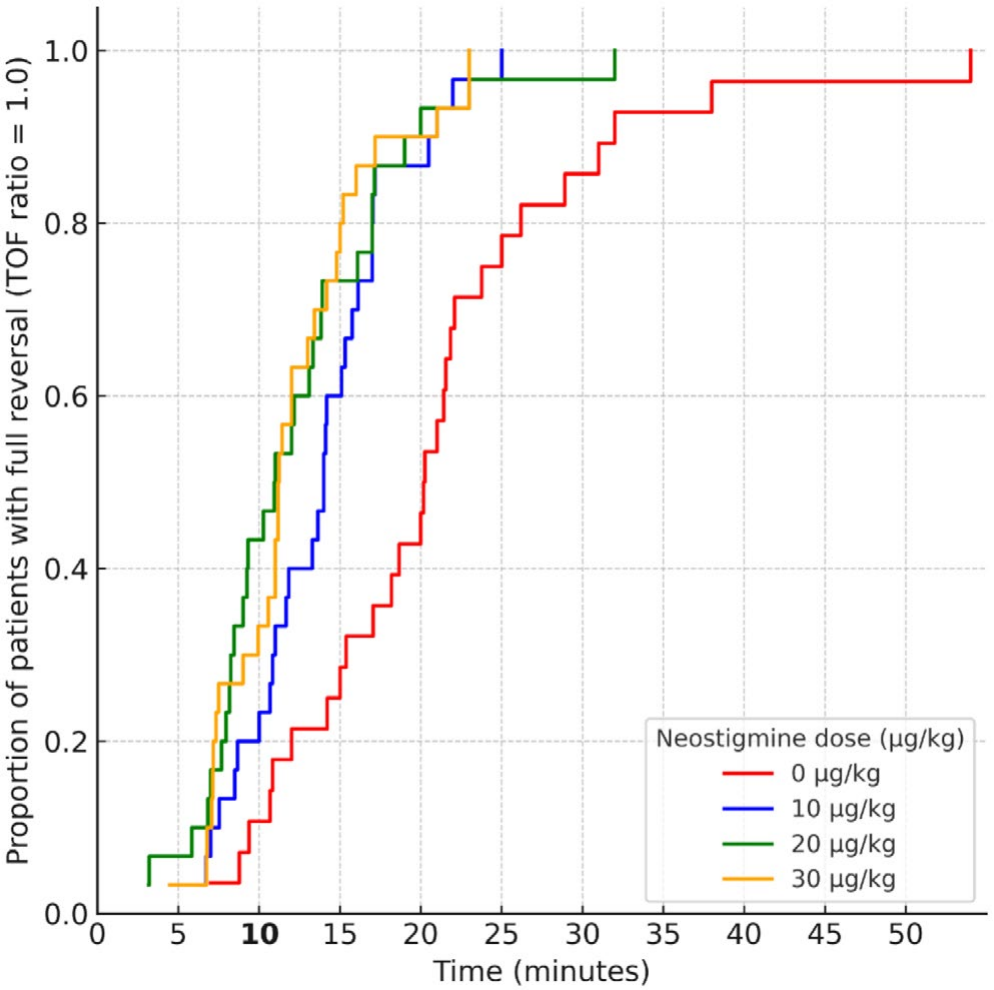
Kaplan–Meier cumulative probability of full reversal.

At 10 min, full reversal had occurred in 10.7%, 23.3%, 43.3%, and 33.3% of patients in the 0, 10, 20, and 30 μg/kg groups, respectively. The cumulative probability of reversal over time is illustrated in Figure 2.

No patient required additional doses of cisatracurium after the initial intubating dose.

Adverse events were uncommon. Eight cases of bradycardia were observed: one patient in the placebo group, two patients in the 10 μg/kg group, three patients in the 20 μg/kg group, and two patients in the 30 μg/kg group. Two cases of tachycardia were observed: one in the 10 μg/kg group and one in the 20 μg/kg group. All episodes of bradycardia were transient and were treated with atropine (20 μg/kg), with prompt resolution and no further clinical consequences. There were no other adverse events.

## 4 Discussion

Our results show that neostigmine effectively accelerates recovery from neuromuscular block in children under total intravenous anesthesia, and that increasing the dose beyond 10 μg/kg does not provide additional clinically meaningful benefit. This is in accordance with the dose–response study by Abdulatif et al. [5], which demonstrated a median effective dose of 7.1 μg/kg to achieve a TOF ratio of 0.8 under volatile anesthesia. After 10 min, which corresponds to the peak effect of neostigmine, fewer than half of the patients in any group had achieved a TOF ratio of 1.0, which is considered clinically unacceptable. Incomplete recovery from neuromuscular blockade has been associated with clinically relevant adverse outcomes, including upper airway obstruction, reintubation, atelectasis, pneumonia, prolonged stay in the postanesthesia care unit, and reduced patient satisfaction [2]. The IQR demonstrated high variability, reinforcing the concept that neuromuscular block should not be assumed and must always be objectively quantified.

Although a TOF ratio of 0.9 is commonly used to define adequate neuromuscular recovery [11], a TOF ratio of 1.0 was chosen as the criterion for full neuromuscular recovery because acceleromyography tends to overestimate the degree of recovery when compared with mechanomyography. Capron et al. [12] demonstrated that an acceleromyographic TOF ratio of 0.9 may still correspond to a mechanomyographic ratio of approximately 0.83, and that only at a ratio of 1.0 can residual paralysis be reliably excluded.

We did not evaluate higher neostigmine doses, such as those commonly recommended for adults, because previous studies have demonstrated a much lower requirement in children. Abdulatif et al. [5] reported that a dose of 5 μg/kg in children was as effective as 50 μg/kg in adults after 10 min. Although current recommendations derived mainly from adult populations favor neostigmine administration at minimal neuromuscular block [11], we investigated reversal at a TOF count of 3, consistent with the lower reversal requirements reported in children. Additionally, our trial was conducted under total intravenous anesthesia, a condition known to reduce the requirement for reversal agents [13]. The low incidence of cholinergic adverse events observed may be partly related to the use of relatively low neostigmine doses combined with proportional atropine administration.

The long‐standing clinical use of neostigmine, together with its low cost and wide availability, makes it a great option for neuromuscular block reversal, provided it is appropriately used. Thilen et al. [7] recently demonstrated, in adults, that more than half of patients at the end of anesthesia are in a superficial block, a scenario where neostigmine is effective. In their protocol, selective administration of neostigmine guided by quantitative monitoring was as safe as sugammadex, with no increase in complications and with substantially lower drug costs. Another large multicenter trial by Beltran et al. [6], in children, found no significant differences in postoperative pulmonary complications between sugammadex and neostigmine, further supporting its use in most clinical situations.

Some limitations of our study should be acknowledged, including its single‐center design and the limited power to detect minor differences. In addition, we used acceleromyographic measurements rather than electromyographic or mechanomyographic techniques, owing to the availability of the former and practical limitations of the latter in the clinical setting. Finally, tonsillectomy is a relatively short procedure and does not invariably require neuromuscular blockade, which may limit the generalizability of our findings.

In conclusion, we found no advantage in using neostigmine doses higher than 10 μg/kg under total intravenous anesthesia when reversal was initiated at a TOF count of 3 in children. These findings emphasize that effective reversal in this setting relies primarily on appropriate timing and quantitative neuromuscular monitoring rather than routine dose escalation. Future studies should explore optimized reversal strategies in pediatric anesthesia, particularly evaluating neostigmine administration at more superficial levels of neuromuscular block.

## Funding

The authors have nothing to report.

## Conflicts of Interest

The authors declare no conflicts of interest.

## Data Availability

The data that support the findings of this study are available from the corresponding author upon reasonable request.
